# Critical Elements of a School Report to Parents on Body Mass Index

**DOI:** 10.5888/pcd12.150165

**Published:** 2015-08-27

**Authors:** Hannah R. Thompson, Jennifer K. Linchey, Kristine A. Madsen

**Affiliations:** Author Affiliations: Jennifer K. Linchey, Kristine A. Madsen, University of California, Berkeley School of Public Health, Berkeley, California.

## Abstract

School-based body mass index (BMI) screening and reporting could have a positive impact on student health, but best practices for writing a report are unknown. Building on previous qualitative work, 8 focus groups were conducted with a diverse group of California parents (n = 79) to elicit feedback on report content and design. Results indicate that parents want a visually appealing, picture-heavy report that clearly defines BMI, avoids stigmatizing language, and includes recommendations for appropriate actions whole families can take. Next steps involve using the final report in a statewide, randomized trial to determine the effectiveness of school-based BMI screening and reporting in reducing childhood obesity.

## Objective

The Institute of Medicine recommends that schools implement BMI screening and reporting practices to combat childhood obesity (1). As of 2010, 20 states, including California, required BMI screening, 9 of which mandated sending reports to parents (2). Current reports are difficult to understand, however, especially for parents with low literacy skills (3). Furthermore, terminology for describing children with a BMI in or higher than the 95th percentile is inconsistent and has provoked controversy, particularly the use of the word “obese.” Despite prior formative research with parents on BMI report design, best practices for reporting remain unknown. This study sought to identify appropriate content for BMI reports, in preparation for a large randomized trial of school-based BMI screening and reporting.

## Methods

Eight focus groups (60–90 minutes) were held in schools with 79 parents (97% female) who identified as Latino, non-Hispanic white, African American, or Asian American; 53% had no more than a high school diploma. Focus groups were conducted for each racial/ethnic category, proportional to the makeup of California public schools: 4 Latino (2 English-speaking, 2 Spanish-speaking); 2 non-Hispanic white; 1 African American; and 1 Asian American. Parents were recruited via school contacts and listservs from 9 low-income elementary and middle schools (prevalence of overweight: 56%; free or reduced-cost meal eligibility: 79%) across 4 Northern California school districts in March through July 2014. One-third of parents reported being told their child had a high BMI.

A semistructured script was used to elicit opinions on 1) preferred language around weight terminology (including “obese” and “overweight,” and prompts for other possible terms); 2) inclusion of BMI (vs height and weight alone); 3) recommendations to promote positive behavior change; and 4) report formatting. Two trained moderators facilitated audio-recorded groups (one led groups in English, one in Spanish) with assistance from a trained co-moderator. The University of California Berkeley Committee for the Protection of Human Subjects approved this study; informed consent was not obtained.

Audio files were transcribed verbatim, and Spanish transcriptions were translated into English. Two researchers coded all transcripts using a codebook developed through a thematic analysis approach (based on predetermined theories about BMI reporting) that allowed for emergent themes (4). Coding and content analysis were executed and organized using NVivo 10.1 software (QSR International).

## Results

An average of 9 parents (range, 4–13) participated per group. Parents had mixed familiarity with BMI, but preferred that it be included (rather than providing height and weight alone) and clearly defined. Parents overwhelmingly wanted a visual representation of BMI, with BMI ranges for each weight category to understand where their child fell.

Parents preferred “at-risk for overweight” and “overweight” to “overweight” and “obese” when describing students in the ≥85th through the >95th and ≥95th percentiles for BMI, respectively. Parents, particularly African American and Latino parents, were opposed to the term “obese,” describing it as “off-putting,” “ugly,” “derogatory,” and “traumatizing” to children. Parents liked “at-risk for overweight” because it “implies that something can be changed.” Parents also preferred “healthy weight” over “normal weight” because “normal” felt vague and unclear.

Parents wanted the report to include recommendations for possible actions to improve their child’s health, especially those relevant for the entire family. Parents were generally familiar with advice to reduce consumption of sugar-sweetened beverages, decrease portion sizes, and limit screen time, but were less familiar with the recommendation for 60 minutes of daily physical activity and wanted that recommendation prominently displayed. When asked to choose, parents preferred the addition of the Healthy Plate Model (5) versus instructions on reading a nutrition label because it is “easier to understand than a nutrition label.”

**Figure 1:**
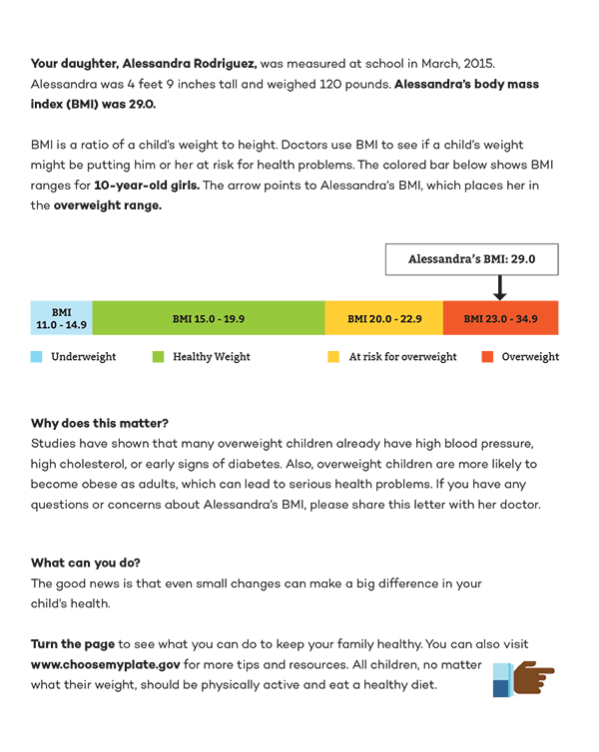
BMI Report (front side of the report)

**Figure 2:**
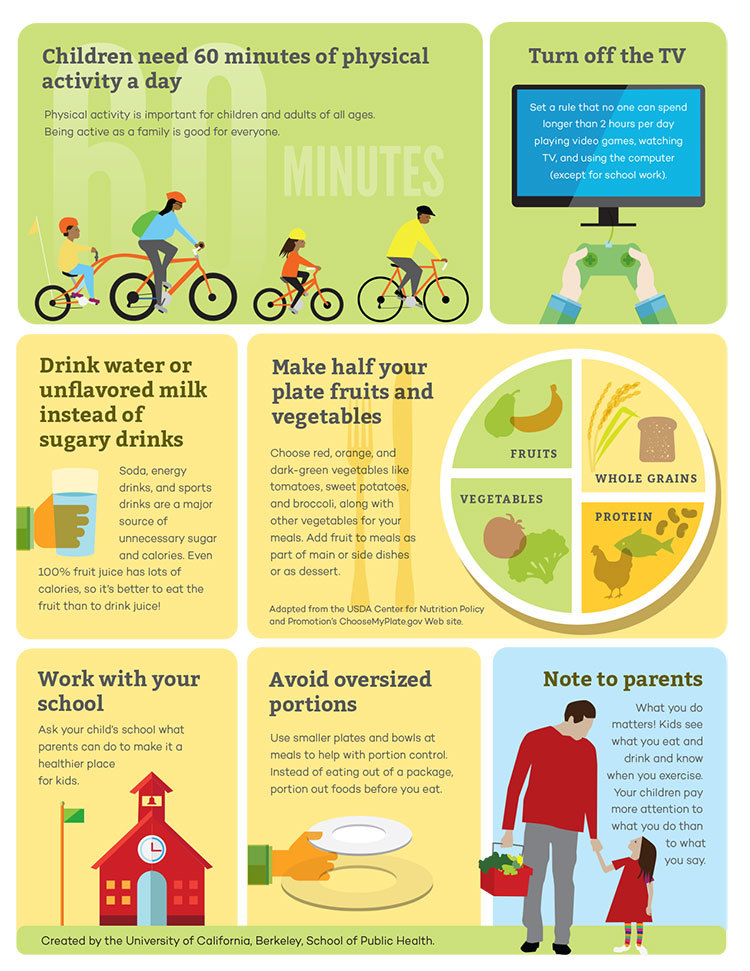
BMI Report (back side of the report).

Parents preferred a short, concise report. Many parents described being visual learners and wanted pictures included: “I think for people who are visual, [pictures are] what draws your eye first. Then you see your child's name, and you see them in the overweight category, and then you think, ‘I'd better read this letter.’” Several parents suggested using stoplight colors (green, yellow, red) to represent the increasing health risks with each BMI category, as well as an arrow to point to exactly where their child falls on the BMI spectrum. Feedback was used to create a single BMI report (Figures 1 and 2).

## Discussion

Building on previous qualitative work (6-8), this study’s feedback from diverse parents led to the development of a new BMI report format. The report reflects parents’ belief that BMI is a more meaningful indicator of weight status than height and weight alone; a simple and clear definition of BMI and interpretable results need to be included; and recommendations should be targeted to the whole family.

Previous formative work (7,9) similarly demonstrated that parents are uncomfortable with the word “obese.” Negative reactions to Massachusetts’ recently implemented BMI report (10) may stem, in part, from the report’s use of the term. Although using “overweight” instead of “obese” is inconsistent with current CDC terminology, the stigma associated with “obese” may so alienate parents that they disregard the report, outweighing potential terminology confusion. CDC itself cites the need to “provide all parents with a clear and respectful explanation of the BMI results” as a BMI measurement program safeguard (11). To assess the potential for unintended consequences related to terminology confusion, we will conduct interviews with parents who receive the BMI report.

On the basis of parents’ feedback and research indicating that pictures help convey key messages to low literacy populations (12), we developed an infographic for the recommended actions. Although prior reports used pictorial representations of children’s weight status (8), previous qualitative work did not address the visual representation of recommendations. The Massachusetts report included pictures (along with heavy text) on physical activity and nutrition tip sheets. The infographic approach allowed us to collapse information into a single page (front and back) that may be more affordable for schools to implement.

Given the widespread use of BMI reporting in schools (2), more evidence on the impact of such reports is critical. The report based on our study may not effectively reduce obesity; however, it will form the basis for a large randomized trial of BMI screening and reporting as a means of reducing pediatric obesity. Results of that study could have important policy implications.
